# Supplementary material for: A TRIM71 binding long noncoding RNA Trincr1 represses FGF/ERK signaling in embryonic stem cells
Source: Nat Commun. 2019 Mar 25;10:1368. doi: 10.1038/s41467-019-08911-w (PMC6433952; doi:10.1038/s41467-019-08911-w)
Supplement: Supplementary file 1 — Supplementary Information [file 41467_2019_8911_MOESM1_ESM.pdf]

Supplementary Information

A TRIM71 binding long noncoding RNA Trincr1 represses FGF/ERK signaling in embryonic stem cells

Authors: Ya-Pu Li[1#], Fei-Fei Duan[1#], Yu-Ting Zhao[1], Kai-Li Gu[1], Le-Qi Liao[1], Huai-Bin Su[1], Jing Hao[1], Kun Zhang[2], Na Yang[2], Yangming Wang[1*]

Affiliations:

1 Beijing Key Laboratory of Cardiometabolic Molecular Medicine, Institute of Molecular Medicine, Peking University, Beijing 100871, China

2 State Key Laboratory of Medicinal Chemical Biology, College of Pharmacy, Nankai University, Tianjin 300353, China

#These authors contributed equally

*Correspondence to: yangming.wang@pku.edu.cn (YW)

Supplementary Figure 1

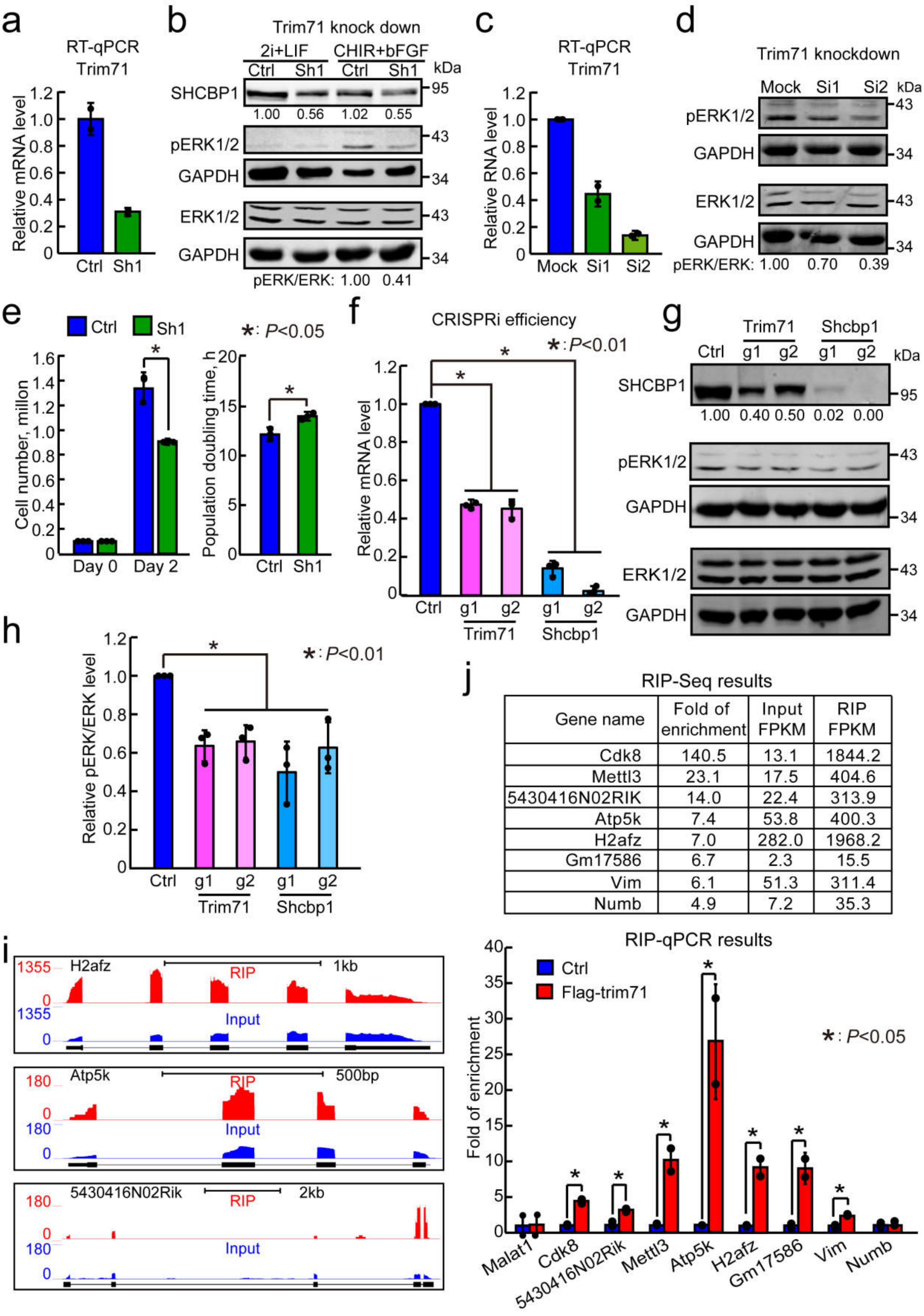

Gene name	Fold of enrichment	Input FPKM	RIP FPKM
Cdk8	140.5	13.1	1844.2
Mettl3	23.1	17.5	404.6
5430416N02RIK	14.0	22.4	313.9
Atp5k	7.4	53.8	400.3
H2afz	7.0	282.0	1968.2
Gm17586	6.7	2.3	15.5
Vim	6.1	51.3	311.4
Numb	4.9	7.2	35.3

Supplementary Figure 1. Trim71 promotes FGF/ERK signaling in ESCs. (**a**) RT-qPCR analysis showing the downregulation of Trim71 by a shRNA. The β-actin gene was used as a control. Data were normalized to the mRNA level of ESCs transfected with control shRNA vectors. n = 2 biological replicates. (**b**) Western blotting analysis of SHCBP1 and phosphorylated ERK (pERK) in Trim71 shRNA ESCs cultured in 2i+LIF or CHIR induced by bFGF. Data are representative of two independent experiments. For quantification of SHCBP1 protein, data were normalized to GAPDH and then to control shRNA vector treated ESCs cultured in 2i+LIF; For quantification of pERK/ERK, data were normalized to GAPDH and then to control shRNA vector treated ESCs cultured in CHIR and induced by 12 ng per ml bFGF. For the analysis of pERK and ERK protein levels throughout this study, two separate gels instead of a single gel were used to avoid unwanted signals due to incomplete stripping. (**c**) RT-qPCR analysis showing the downregulation of Trim71 by two sets of siRNAs. The β-actin gene was used as a control. Data were normalized to the mRNA level of mock transfected ESCs. n = 2 biological replicates. (**d**) Western blotting analysis of phosphorylated ERK induced by bFGF in ESCs treated with two different sets of Trim71 siRNAs. For quantification of pERK/ERK, data were normalized to GAPDH and then to mock transfected wild type ESCs. (**e**) Growth (left) and population doubling time (right) for control and Trim71 shRNA knockdown ESCs in 2i+LIF medium. For the cell growth experiment, 0.2 million cells were plated in a well of 12-well plate and grown for two days before counting. Representative data of three independent experiments are shown. Cell population doubling time was obtained using equation

$N_{end}=N_{start}\times2^{(t/T)}$. T is the cell population doubling time, N_{start} is the starting number of ESCs plated, N_{end} is the ending number of ESCs after growing for a period of time (t). n = 3 biological replicates. (**f**) RT-qPCR analysis of knockdown efficiency of CRISPRi constructs targeting Trim71 and Shcbp1. The β-actin gene was used as a control. Data were normalized to control gRNA treated ESCs. n = 3 biological replicates. (**g**) Western blotting analysis of SHCBP1 and phosphorylated ERK induced by bFGF in ESCs treated with control, Trim71 and Shcbp1 CRISPRi gRNAs. (**h**) Quantification of pERK/ERK in (**g**). For quantification of pERK/ERK, data were normalized to GAPDH and then to control gRNA treated ESCs cultured in CHIR and induced by 12 ng per ml bFGF. n = 3 independent experiments. (**i**) RIP-Seq tracks at H2afz, Atp5 and 5430416N02RIK loci. Shown are normalized read counts per million. FLAG-TRIM71-RIP and input were scaled to the same level. (**j**) RIP-seq data and RT-qPCR analysis of 8 randomly selected candidates. Top panel: fold enrichment of candidates in RIP-seq; bottom panel: RT-qPCR analysis of candidates. Data were normalized to ESCs transfected with control empty overexpression vectors. n = 2 biological replicates. Shown are mean ± SD for all panels. For (e) and (j), *P* values were determined by unpaired two-sided Student's *t*-test. For (f) and (h), *P* values were determined by unpaired one-way ANOVA with two-sided Dunnett's test.

Supplementary Figure 2

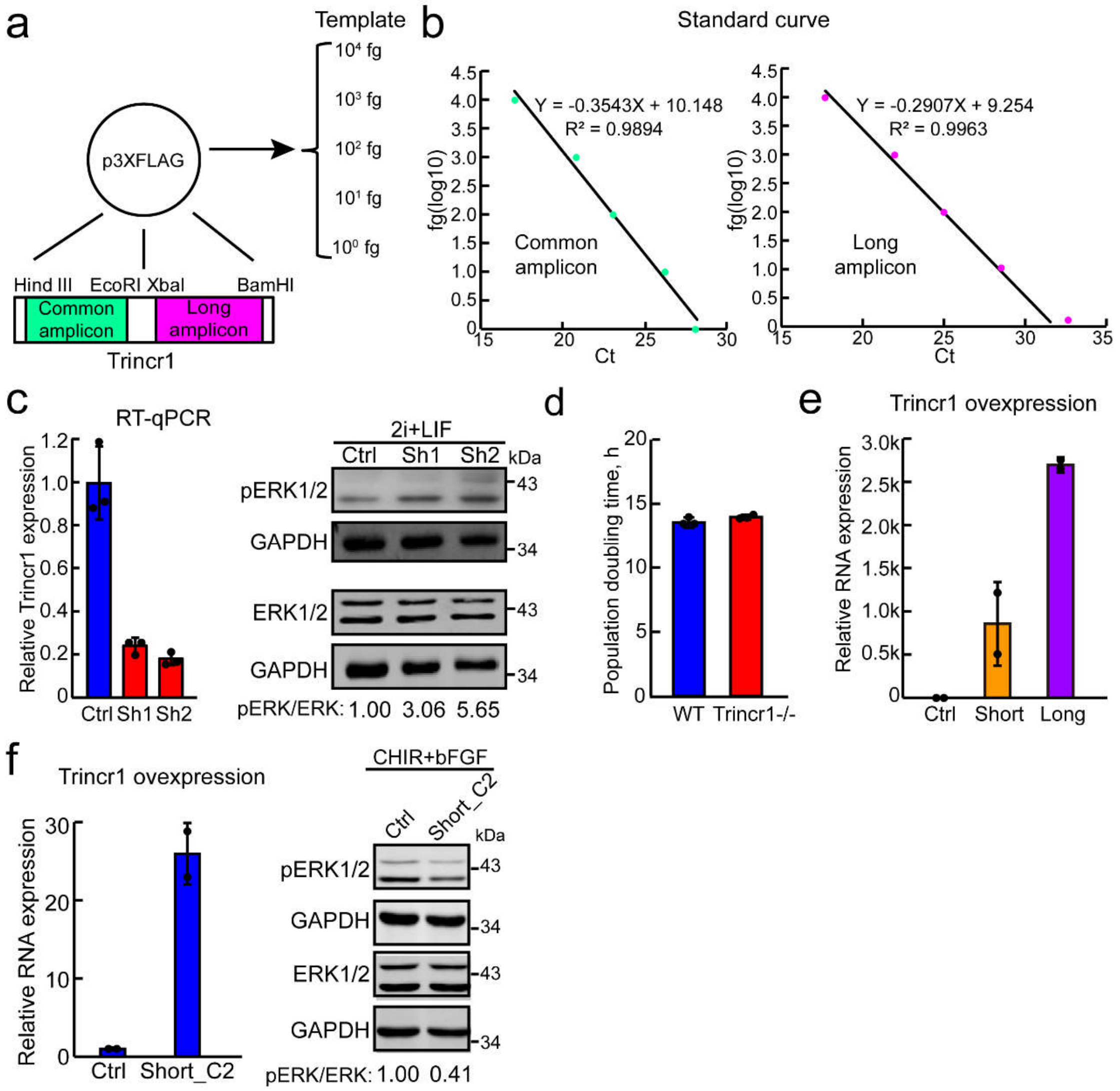

Supplementary Figure 2. Characterization of Trincr1. (**a**) Schematic design for quantifying relative level of Trincr1_L and S. Both the common (L plus S) and long isoform (Trincr1_L) amplicons were cloned in tandem into the same plasmid. Five different dilutions were made for qPCR to make the standard curve. The amount of Trincr1_L and total Trincr1 was calculated by fitting the Ct value into the respective standard curve. The amount of Trincr1_S was calculated by subtracting the value of Trincr1_L from the value of total Trincr1. Shown are representative standard curves (**b**) for the common and long isoform amplicons. (**c**) Western blotting analysis of basal pERK level in Trincr1 shRNA ESCs. Left: RT-qPCR showing knock down of Trincr1 by two sets of shRNAs. The β-actin gene was used as a control. Data were normalized to control empty shRNA vector treated ESCs. n = 3 biological replicates. Right: Western blotting of pERK and ERK. For pERK/ERK, data were normalized to GAPDH and then to control empty shRNA vector treated ESCs cultured in 2i+LIF. **(d)** Population doubling time for wild-type and *Trincr1-/-* ESCs in 2i+LIF medium. n = 3 biological replicates. **(e)** RT-qPCR analysis showing the level of Trincr1 in Trincr1 overexpression ESCs. n = 2 biological replicates. **(f)** Western blotting analysis of bFGF induced phosphorylated ERK in ESCs with low level overexpression of Trincr1 short isoform. Left: RT-qPCR showing overexpression of Trincr1. n = 2 biological replicates. Right: Western blotting of pERK and ERK. For pERK/ERK, data were normalized to GAPDH and then to control empty overexpression vector treated ESCs cultured in CHIR and induced by 12 ng per ml bFGF. For RT-qPCR in (e) and (f), the β-actin gene

was used as a control. Data were normalized to control empty overexpression vector treated ESCs. Shown are mean ± SD for all panels.

Supplementary Figure 3

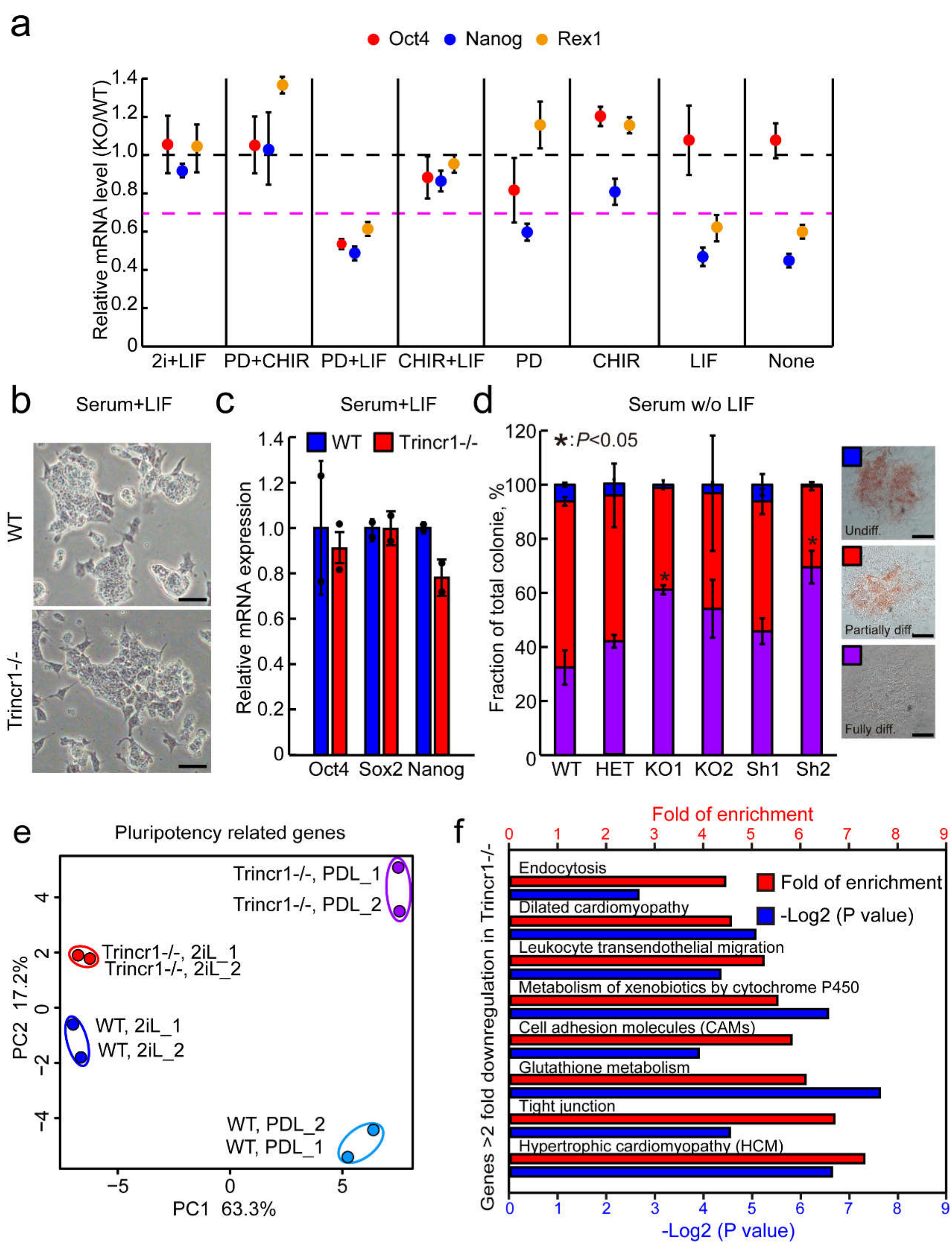

Supplementary Figure 3. Trincr1 promotes the self-renewal and the expression of pluripotency genes. (**a**) RT-qPCR analysis of key pluripotency genes in *Trincr1-/-* ESCs in different media contexts. The β-actin gene was used as a control. For each gene, data were normalized to the mRNA level of wild type ESCs cultured in the same medium. n = 2 biological replicates. (**b**) Morphology of *Trincr1-/-* ESCs in conventional serum+LIF culture. Scale bars, 50 μm. (**c**) RT-qPCR analysis of Oct4, Sox2 and Nanog in *Trincr1-/-* ESCs in conventional serum+LIF medium. The β-actin gene was used as a control. For each gene, data were normalized to the mRNA level of wild type ESCs. n = 2 biological replicates. (**d**) Fraction of un-, partially and fully differentiated colonies based on alkaline phosphatase staining for ESCs with different genotypes in serum medium without (w/o) LIF supplement. HET: *Trincr1-/+* ESC; KO1 and KO2: two independent *Trincr1-/-* ESC clones; sh1 and sh2: Trincr1 shRNA ESC clones with two different sets of shRNAs. Representative alkaline phosphatase staining images are shown. Scale bars, 200 μm. n = 2 biological replicates. **(e)** Principal component analysis of selected pluripotency related genes in wild-type and *Trincr1-/-* ESCs in 2i+LIF and PD+LIF. (**f)** KEGG pathway analysis of downregulated genes in *Trincr1-/-* ESCs in PD+LIF. Top 8 enriched pathways are shown with *P* values. Shown are mean ± SD for all panels. For (d), *P* values were determined by unpaired two-way ANOVA with two-sided Dunnett's test. For (f), Gene Ontology analysis was performed by DAVID v6.8. Fisher's exact test was used to determine *P* value.

Supplementary Figure 4

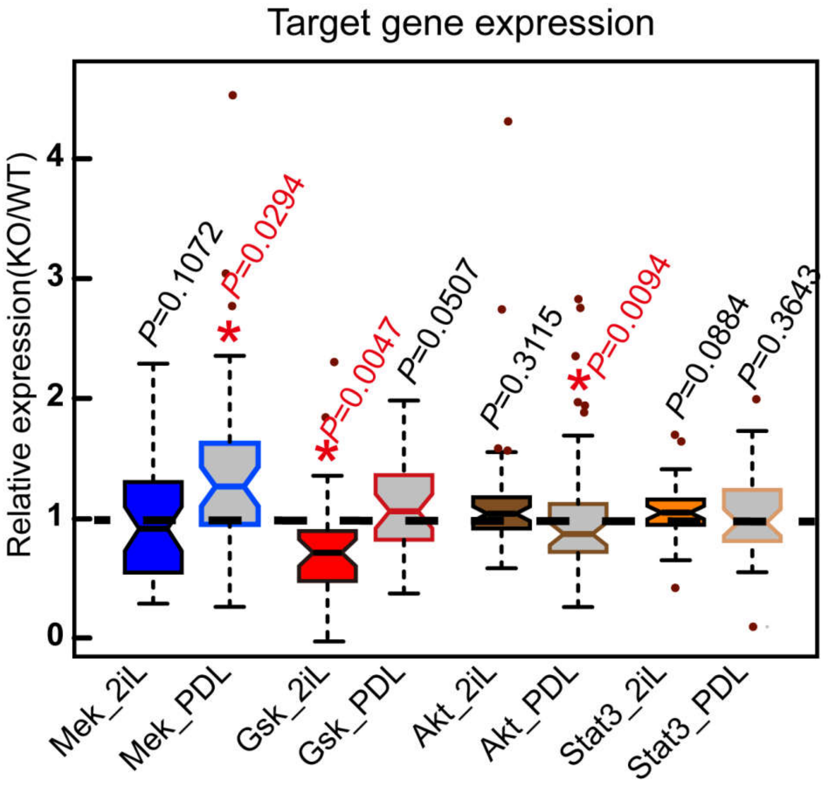

Supplementary Figure 4. Trincr1 represses the expression of ERK target genes. Pathway analysis for wild type and *Trincr1-/-* ESCs in 2i+LIF or PD+LIF medium. Boxes indicate the 25th to 75th percentiles and the central bar represents the median. *P* values were determined by two-tailed Wilcoxon signed-rank test.

Supplementary Figure 5

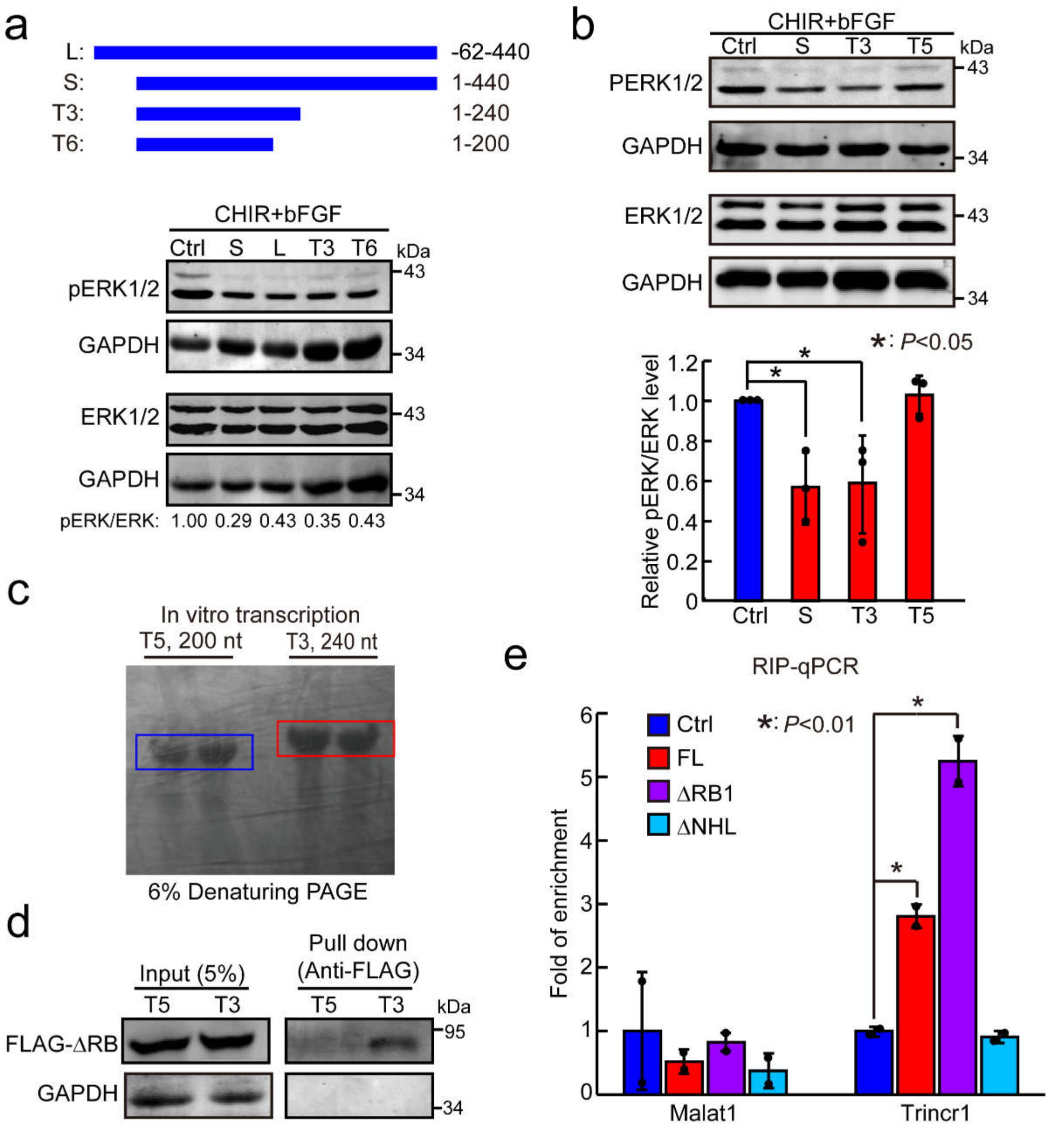

Supplementary Figure 5. Trincr1 physically interacts with TRIM71. (**a**) Western blotting analysis of phosphorylated ERK in ESCs overexpressing Trincr1_S, Trincr1_L and two truncated constructs of Trincr1 induced by bFGF. (**b**) Western blotting analysis of phosphorylated ERK in control and Trincr1_S or truncated T3 or T5 overexpressing ESCs induced by bFGF. For quantification of pERK/ERK, data were normalized to GAPDH and then to control overexpression vector transfected ESCs cultured in CHIR and induced by 12 ng per ml bFGF. n = 3 independent experiments. (**c**) Urea denaturing PAGE purification of in vitro transcribed T3 and T5. The RNA products were then used to pull down protein factors for mass spectrometry analysis or western blotting analysis. (**d**) Western blotting analysis of Flag tagged, RING and B1 domain truncated TRIM71 pulled down by T3 and T5. Shown is a representative gel of two independent experiments. (**e**) RT-qPCR analysis of Trincr1 and Malat1 following Flag RIP in control, Flag-Trim71, Flag-RING and B1 truncated Trim71, and Flag-NHL domain truncated Trim71 overexpressing ESCs. Data were normalized to control 3X FLAG overexpression vector transfected ESCs. n = 2 biological replicates. Shown are mean ± SD for (b) and (e). For (b) and (e), *P* values were determined by unpaired one-way and two-way ANOVA with two-sided Dunnett's test, respectively.

Supplementary Figure 6

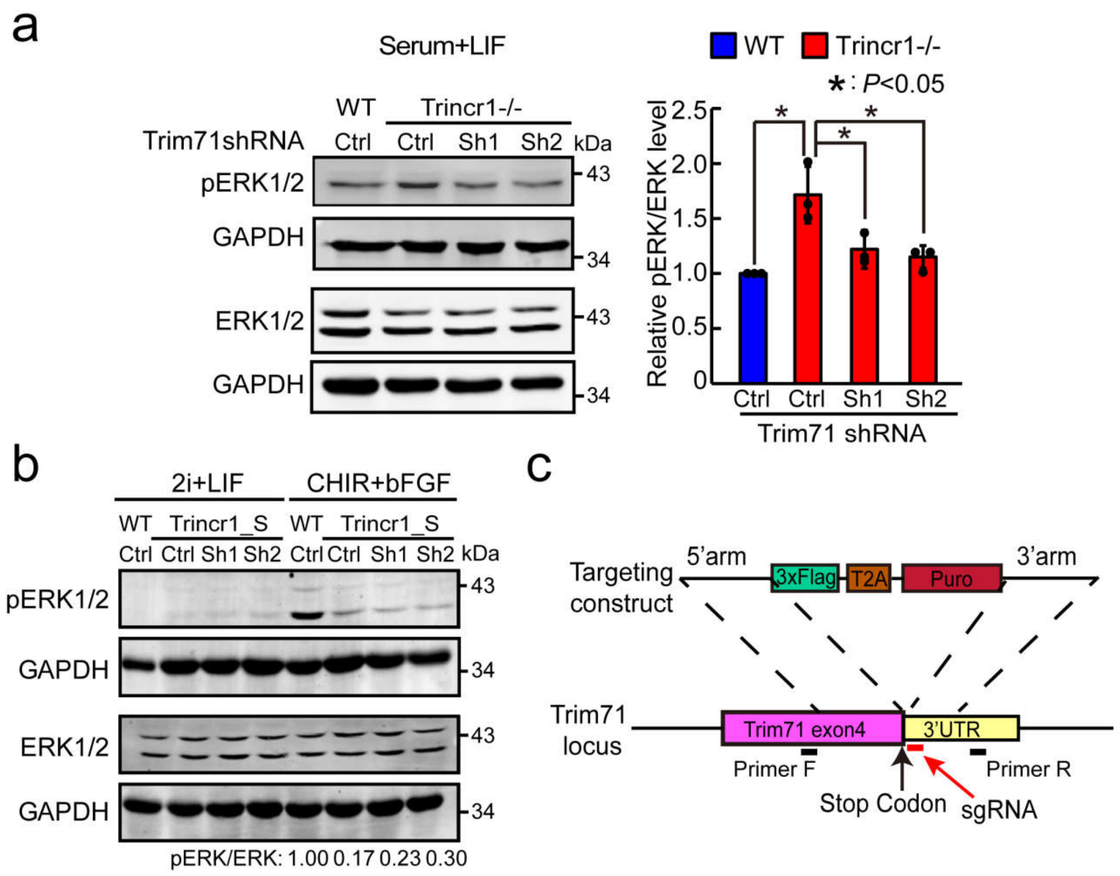

Supplementary Figure 6. Trincr1 represses FGF/ERK signaling through Trim71. (**a**) Western blotting analysis of pERK in serum+LIF cultured wild type ESCs transfected with control shRNA vectors and *Trincr1-/-* ESCs transfected with control and Trim71 shRNA vectors. Sh1 and Sh2 are two different sets of shRNA constructs. Shown are representative gel images. Quantification of pERK/ERK were normalized to wild type ESCs transfected with control shRNA vectors. n = 3 independent experiments. (**b**)Western blotting analysis of pERK in control or Trim71 shRNA ESCs in Trincr1_S overexpression background. For quantification of pERK/ERK, data were normalized to GAPDH and then to empty control ESCs cultured in CHIR and induced by 12 ng per ml bFGF. (**c**) Graphic illustration showing 3X FLAG-knockin strategy to detect endogenous TRIM71 protein. The red arrow indicates the location of the gRNA, the black arrow indicates the location of TIRM71 stop codon. Primers for genomic PCR characterizing knock in allele are shown. For (a), shown are mean ± SD. *P* values were determined by unpaired one-way ANOVA with two-sided Dunnett's test.

Supplementary Figure 7

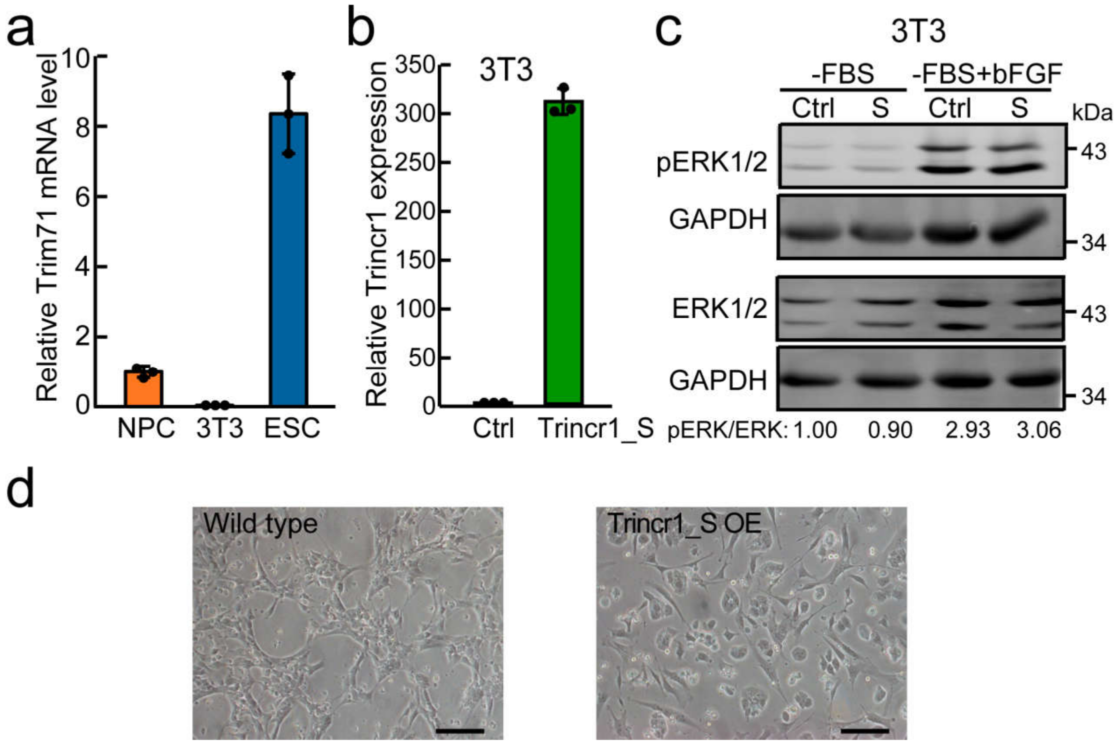

Supplementary Figure 7. Ectopic expression of Trincr1 represses FGF/ERK signaling in NPCs. (**a**) RT-qPCR analysis of Trim71 in NPCs, 3T3 and ESCs. The β-actin gene was used as a control. Data were normalized to NPCs. n = 3 biological replicates. (**b**) RT-qPCR analysis of Trincr1 in empty control or Trincr1 overexpression 3T3 cells. The β-actin gene was used as a control. Data were normalized to control overexpression vector treated 3T3 cells. n = 3 biological replicates. (**c**) Western blotting analysis of pERK in 3T3 cells overexpressing Trincr1_S. For quantification of pERK/ERK, data were normalized to GAPDH and then to empty vector transfected 3T3 cells cultured in serum free media. (**d**) Morphology of wild type and Trincr1_S overexpressing NPCs. Scale bars, 100 μm. Shown are mean ± SD for (a) and (b).

Supplementary Figure 8. Uncropped western blot images for the main figures

Fig. 2e

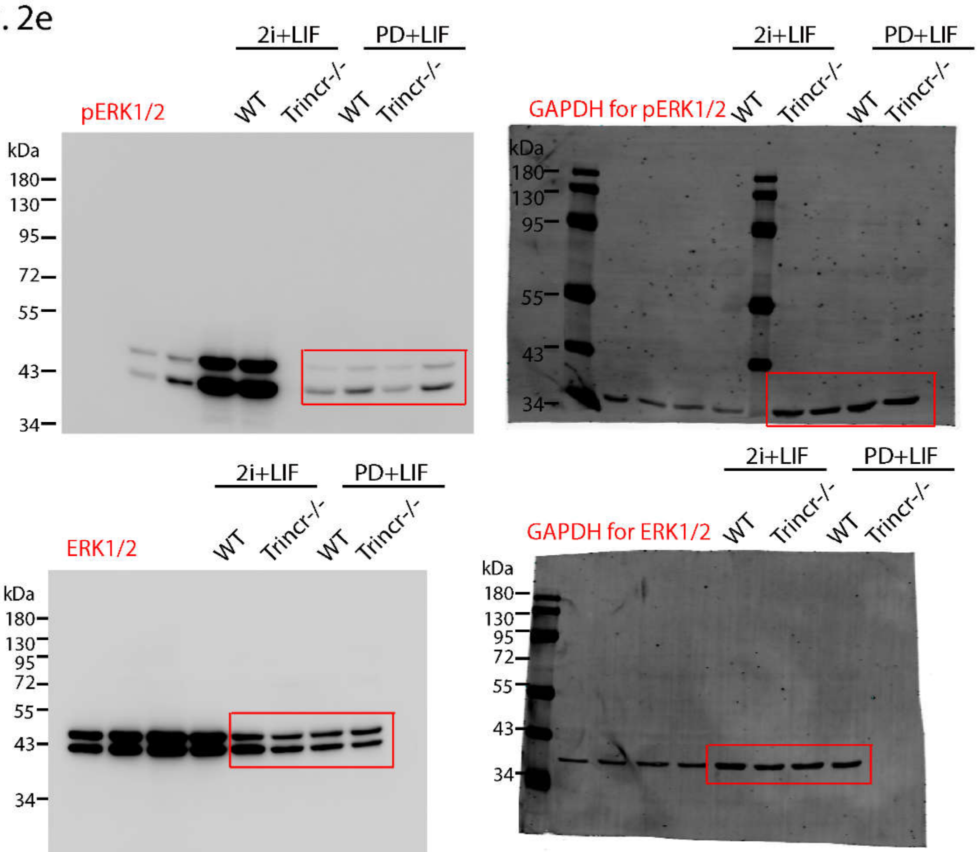

Fig. 2f

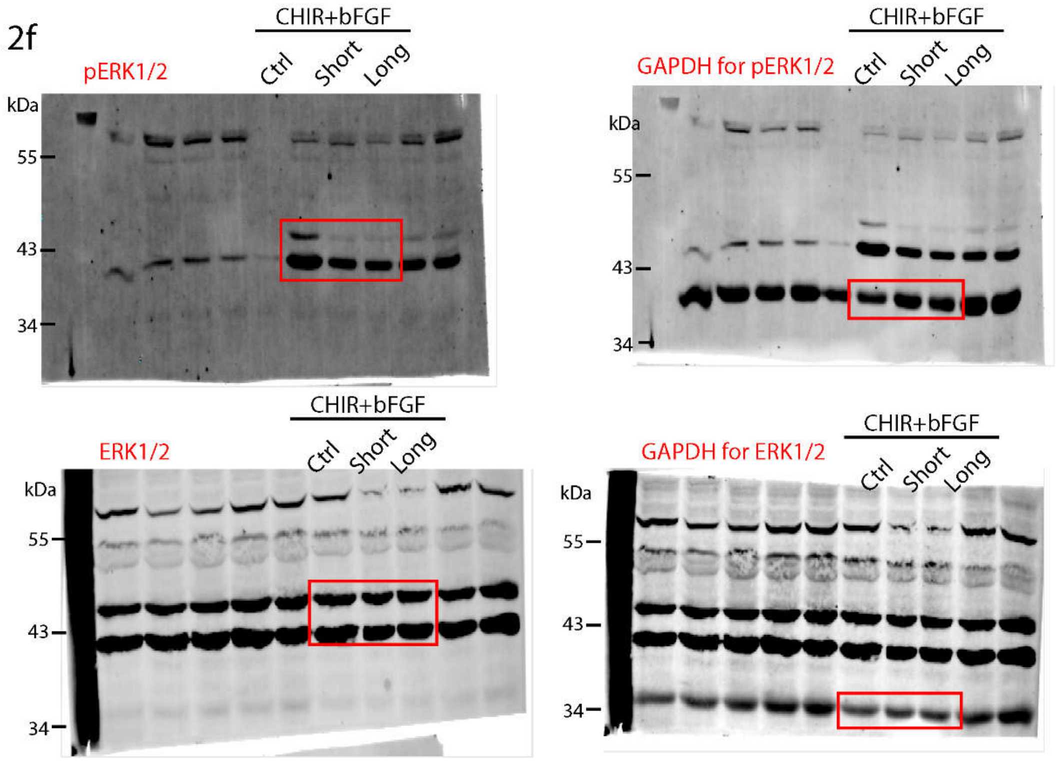

Fig. 4d

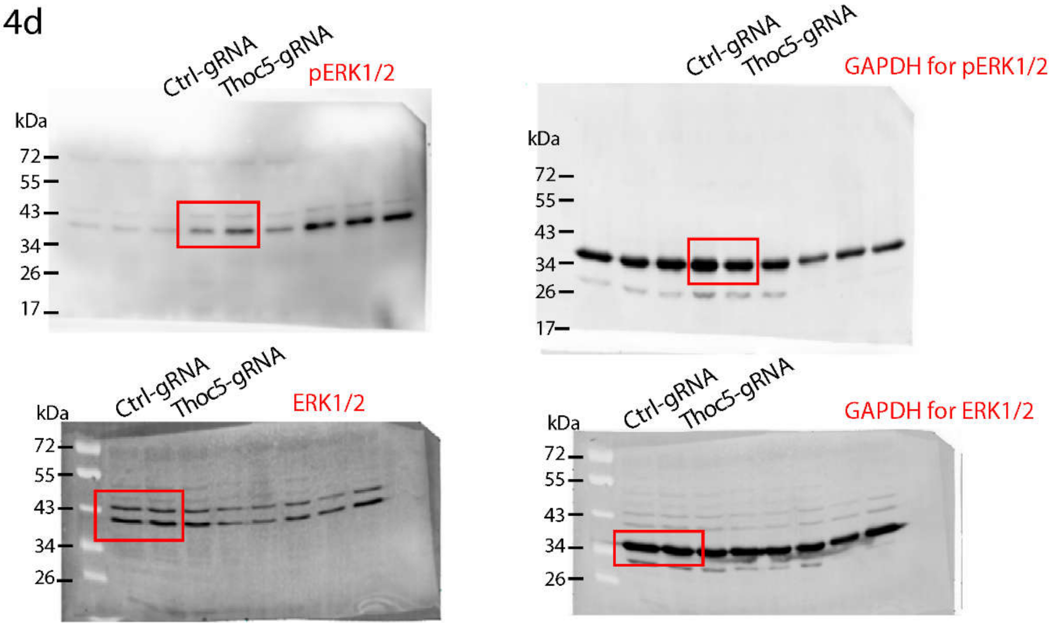

Ctrl-gRNA
Thoc5-gRNA
pERK1/2
kDa
72
55
43
34
26
17
GAPDH for pERK1/2
ERK1/2
GAPDH for ERK1/2

Fig. 5b

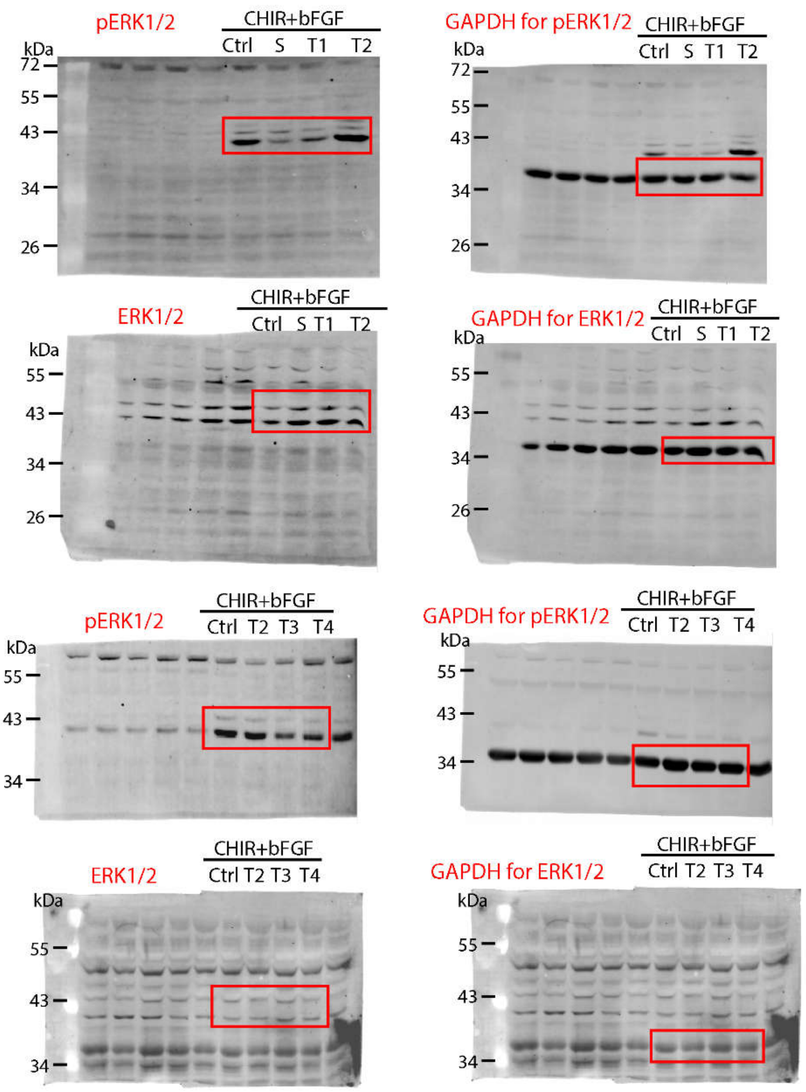

Fig. 5e

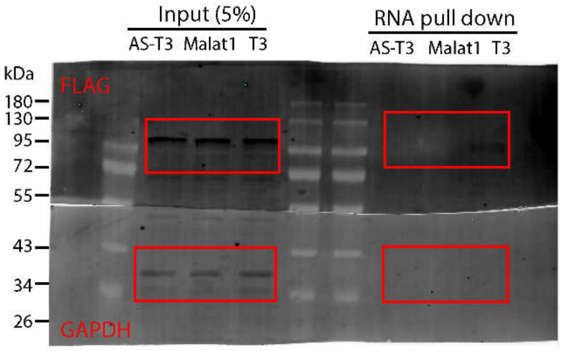

Fig. 5g

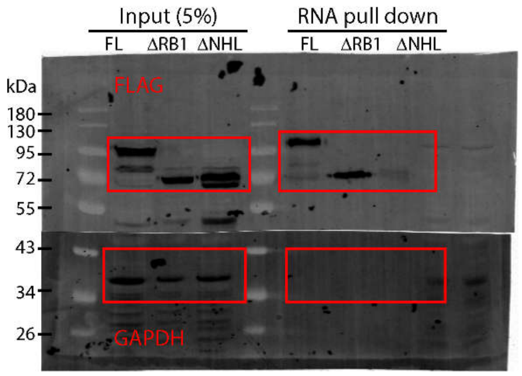

Fig. 6b

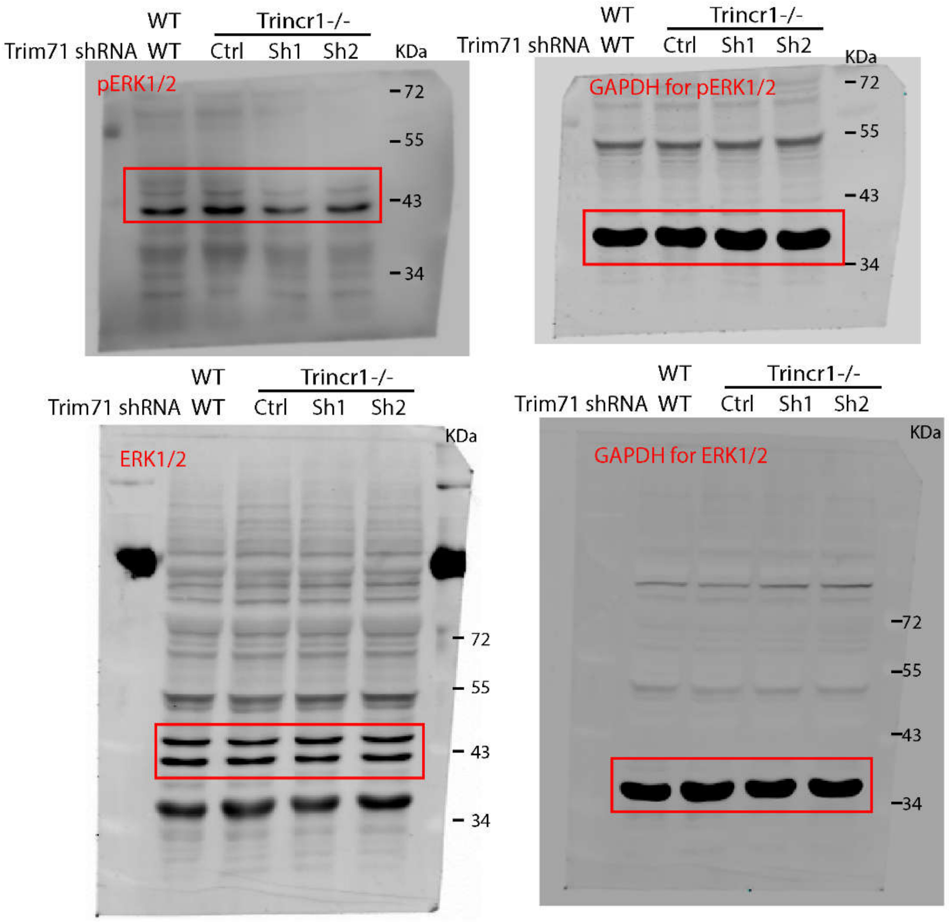

Fig. 6f

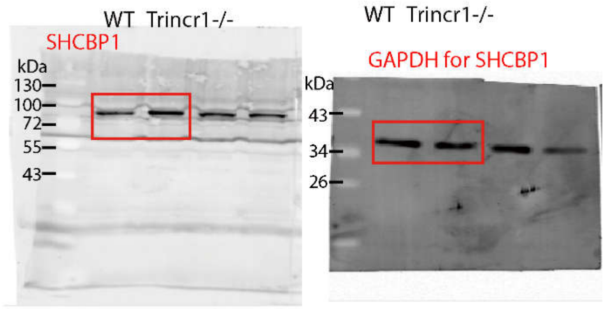

Fig. 6h

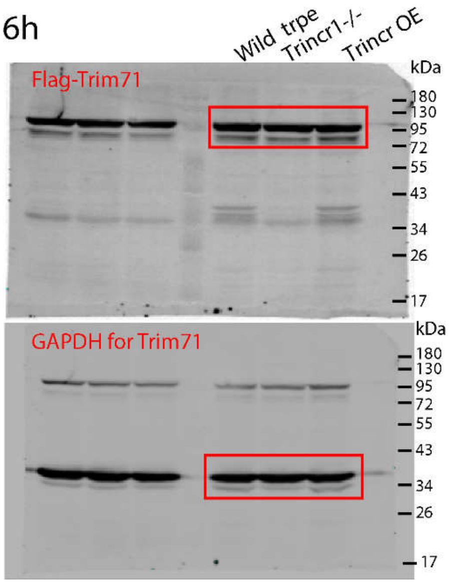

Fig. 6i

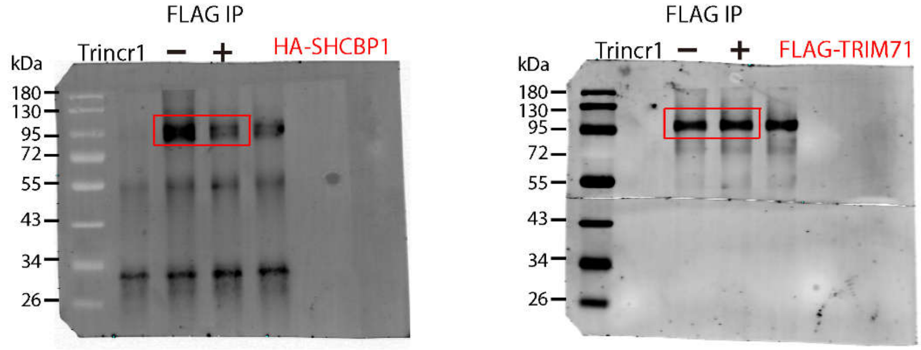
FLAG IP
Trincr1 − +
HA-SHCBP1
kDa
180
130
95
72
55
43
34
26
FLAG IP
Trincr1 − +
FLAG-TRIM71
kDa
180
130
95
72
55
43
34
26

Fig. 7a

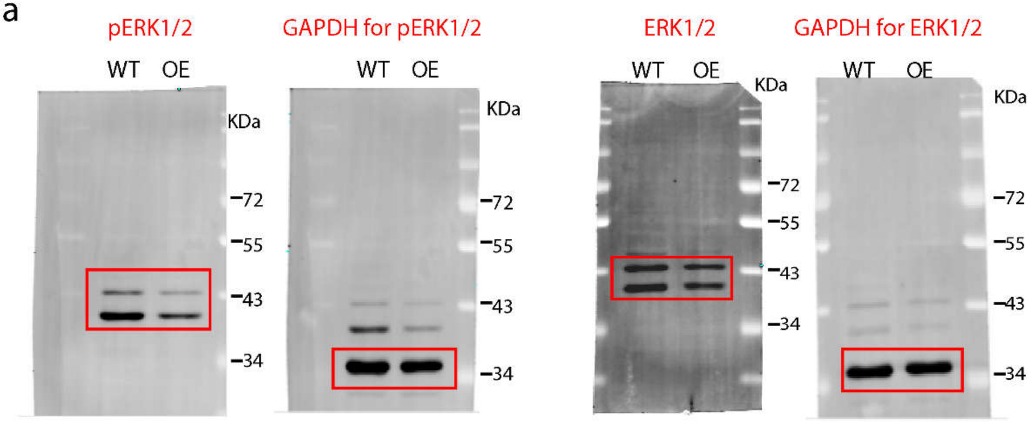
pERK1/2
WT OE
KDa
72
55
43
34
GAPDH for pERK1/2
WT OE
KDa
72
55
43
34
ERK1/2
WT OE
KDa
72
55
43
34
GAPDH for ERK1/2
WT OE
KDa
72
55
43
34

Supplementary Table 1 Sequence of RT-qPCR primers

Gene	Primer	Sequence (5'-3')
Actb	S1F	CCACCATGTACCCAGGCATT
	S1R	CCGATCCACACAGAGTACTT
Gapdh	S1F	CAAGGTCATCCATGACAACT
	S1R	GTCTTCTGGGTGGCAGTGAT
Oct4	S1F	CACCCTGGGCGTTCTCTTT
	S1R	ATGTTCTTAAGGCTGAGCTGCAA
Sox2	S1F	GCAAGTACTGGCAAGACCGTT
	S1R	CGATATCAACCTGCATGGACAT
Nanog	S1F	GCTCAGCACCAGTGGAGTATCC
	S1R	TCCAGATGCGTTCACCAGATAG
Rex1	S1F	ATCGCTGTGGGCATTAGAGA
	S1R	CGATCCTGCTTTCTTCTGTGT
Klf2	S1F	GGTCCCCTTGCAAACAGACT
	S1R	CAGAACTGGTGGCAGAGTCA
Esrrb	S1F	TACCTGAACCTGCCGATTTC
	S1R	CCCAGTTGATGAGGAACACA
Tbx3	S1F	TCCGGGCTCCACTTGAAAT
	S1R	CCCCTACTCACACAACAATCTCAAC
Trim71	S1F	GGCCAATGGCAGCTTCCT
	S1R	CCACCACGACGATCAATCC
Trincr1	S1F	CGAGCCTTCTACTTGGCCAA
	S1R	CTTCAGGACATGCTTGGTAACT
Trincr1 long	S1F	GGAGTCAGACGAGGGAGCGC
	S1R	GCAGCTGTGTTGGCCAAGTAG
Fgf5	S1F	TGAACCCTGCCACTTACTGAAA
	S1R	TCATCACATTCCCGAATTAAGCT
U1	S1F	CTGGCAGGGGAGATACCATG
	S1R	AGTCGAGTTTCCCGCATTTG
Malat1	S1F	GGTCTCCCCACAAGTGATTTCT
	S1R	GGGTGAAGGGTCTGTGATTAGG
Shcbp1	S1F	CTTTTGTGGATTGCACGGGT
	S1R	TTCCAGTGTGGTCTTGCCAT

Thoc5	S1F	GGCCTGTGATAAGACGCTGT
	S1R	CCTTGCGCTTGTCATCCAAC
Thoc2	S1F	CTCCACAGTAAAGGACAGTCTCA
	S1R	TGGACAGTGGTGGAGGGTTA
Nxf1	S1F	GTCCAGCTATATCAGCGCCA
	S1R	GGGGCTTCGACATCAAAGGA
Aly	S1F	AGAGGCGGCATGACAAGAAA
	S1R	AGCTGCTGCTTTGAGTTCCT
Nestin	S1F	GACTCTGGAATGCAAGGGCT
	S1R	GCTGCCTGTAGACCCTCATC
Pax6	S1F	CACATGAACAGTCAGCCCAT
	S1R	CTTGGACGGGAACTGACACT
Clu	S1F	GCTGTTTGACTCTGACCCCA
	S1R	TCTATCTCATTCCGCACGGC
Gfap	S1F	CAAGCACGAAGCTAACGACT
	S1R	CGCTCTTCCTGTTCGCGCAT
Aqp4	S1F	ACATGGAGGTGGAGGACAAC
	S1R	TTCTCCACGGTCAATGTCAA
Cdk8	S1F	GTGCTAGGGCGAGCATTTG
	S1R	GTCAGATCACGCTGTTACACTCAA
5430416N02Rik	S1F	TCTGGCATCATGTCTACGCC
	S1R	AGCTCGGTTTCCACCTCTCT
Mettl3	S1F	CCAGATGAAATATATGGCATGATTGA
	S1R	CACATTGTGTGGTCGTCCAAA
Atp5k	S1F	CTCATCAAGTTCGGCCGGTA
	S1R	TCCTCCGCTGCTATTCTCCT
H2afz	S1F	ATCTAGGACAACCAGCCACG
	S1R	GACGAGGGGTGATACGCTTT
Gm17586	S1F	GACCGAGGTCTATGGGAGGT
	S1R	GTGGTGACGCTCATCTGTGA
Vim	S1F	CCAGATGAAATATATGGCATGATTGA
	S1R	CACATTGTGTGGTCGTCCAAA
Numb	S1F	CAGATCACCAGTGCCTTCAGC
	S1R	CCACTCAGTCCCTTGTAACACAGG

Supplementary Table 2 Target sequence of siRNAs, shRNAs and CRISPR i sgRNAs

Gene	si/sh/sgRNA	Sequence (5'-3')
Trim71	si1	CAAGAAGAUGACCGCAUUA
	si2	GGGCAAGAUCCUCGUUUCA
Trincr1	sh1	GGCAAAGCCACACCAUAUAUC
	sh2	AGAGGUCCAAAGCACGUAAAC
Trim71	sh1	GUACUUACAUAGUGAGCUA
	sh2	GGAUCAUAGUGGCCGACAA
Trim71	sgRNA1	ATAACCCGCGTTCTGAGAGG
	sgRNA2	CTCGGCGAATCGTTCGTAGG
Shcbp1	sgRNA1	TGAAATGGCTGATGATTTGC
	sgRNA2	AAGGACGCGATTACCTGGGT
Thoc5	sgRNA	AAACGCTCCTGGGTGGCGGT
Thoc2	sgRNA	TGCGGGAACCACCACAGCCG
Nxf1	sgRNA	CACCGCTACCTACGGACGTA
Aly	sgRNA	GCCGCCTCGATTCACCCGCG
