# Supplementary material for: A TRIM71 binding long noncoding RNA Trincr1 represses FGF/ERK signaling in embryonic stem cells
Source: Nat Commun. 2019 Mar 25;10:1368. doi: 10.1038/s41467-019-08911-w (PMC6433952; doi:10.1038/s41467-019-08911-w)
Supplement: Supplementary file 3 — Description of Additional Supplementary Files [file 41467_2019_8911_MOESM3_ESM.pdf]

Description of Additional Supplementary Files

Supplementary Data 1

Trim71 RIP sequencing data and list of Trim71 binding RNA.

Supplementary Data 2

Gene expression in wild type and *Trincr1*-/- ESCs in 2i+LIF and PD+LIF.
