# Supplementary material for: A TRIM71 binding long noncoding RNA Trincr1 represses FGF/ERK signaling in embryonic stem cells
Source: Nat Commun. 2019 Mar 25;10:1368. doi: 10.1038/s41467-019-08911-w (PMC6433952; doi:10.1038/s41467-019-08911-w)
Supplement: Supplementary file 6 — Reporting Summary [file 41467_2019_8911_MOESM6_ESM.pdf]

nature research

Corresponding author(s): Yangming Wang

Last updated by author(s): Jan 21, 2019

Reporting Summary

Nature Research wishes to improve the reproducibility of the work that we publish. This form provides structure for consistency and transparency in reporting. For further information on Nature Research policies, see Authors & Referees and the Editorial Policy Checklist.

Statistics

For all statistical analyses, confirm that the following items are present in the figure legend, table legend, main text, or Methods section.

n/a	Confirmed	
☐	☒	The exact sample size (n) for each experimental group/condition, given as a discrete number and unit of measurement
☐	☒	A statement on whether measurements were taken from distinct samples or whether the same sample was measured repeatedly
☐	☒	The statistical test(s) used AND whether they are one- or two-sided<br>*Only common tests should be described solely by name; describe more complex techniques in the Methods section.*
☐	☒	A description of all covariates tested
☐	☒	A description of any assumptions or corrections, such as tests of normality and adjustment for multiple comparisons
☐	☒	A full description of the statistical parameters including central tendency (e.g. means) or other basic estimates (e.g. regression coefficient) AND variation (e.g. standard deviation) or associated estimates of uncertainty (e.g. confidence intervals)
☐	☒	For null hypothesis testing, the test statistic (e.g. F, t, r) with confidence intervals, effect sizes, degrees of freedom and P value noted<br>*Give P values as exact values whenever suitable.*
☒	☐	For Bayesian analysis, information on the choice of priors and Markov chain Monte Carlo settings
☒	☐	For hierarchical and complex designs, identification of the appropriate level for tests and full reporting of outcomes
☒	☐	Estimates of effect sizes (e.g. Cohen's d, Pearson's r), indicating how they were calculated

Our web collection on statistics for biologists contains articles on many of the points above.

Software and code

Policy information about availability of computer code

Data collection	qRT-PCR data were collected using the Applied Biosystems StepOne Softeware v2.2. Western blot data were collected using Oddssey and Amersham imager 600.
Data analysis	GraphPad Prism6 were used to calculate means, standard deviation, P value and to perform statistical analyses. Fiji Image J was used for imaging analysis. RNA-seq analysis: Sequenced reads were mapped to mm9 whole genome using STAR v2.5.0. Fragments Per Kilobase of Transcript Per Million Fragments (FPKM) were calculated by Cufflinks v2.2.1. GSEA was performed using GSEA v2.4 software with FPKM data. R 3.1.1 was used for the generation of boxplot. GO Enrichment Analysis of differential expression genes was performed with DAVID v6.8.

For manuscripts utilizing custom algorithms or software that are central to the research but not yet described in published literature, software must be made available to editors/reviewers. We strongly encourage code deposition in a community repository (e.g. GitHub). See the Nature Research guidelines for submitting code & software for further information.

Data

Policy information about availability of data

All manuscripts must include a data availability statement. This statement should provide the following information, where applicable:

- Accession codes, unique identifiers, or web links for publicly available datasets
- A list of figures that have associated raw data
- A description of any restrictions on data availability

All data generated or analyzed during this study are included in the manuscript and its supplementary information files. RNA-seq and RIP-seq data are deposited in NCBI's Gene Expression Omnibus under the accession GSE125458[https://www.ncbi.nlm.nih.gov/geo/query/acc.cgi?acc=GSE125458]. Proteomics data can be accessed on PRIDE (PRoteomics IDEntifications) database under the accession number: PXD012493[http://proteomecentral.proteomexchange.org/cgi/GetDataset?ID=PXD012493]. All data that support the findings of this study are available from the corresponding authors upon request.

Field-specific reporting

Please select the one below that is the best fit for your research. If you are not sure, read the appropriate sections before making your selection.

☒ Life sciences ☐ Behavioural & social sciences ☐ Ecological, evolutionary & environmental sciences

For a reference copy of the document with all sections, see nature.com/documents/nr-reporting-summary-flat.pdf

Life sciences study design

All studies must disclose on these points even when the disclosure is negative.

Sample size	No sample- size calculation was performed.
Data exclusions	No data was excluded from experiments and analysis.
Replication	Most data were repeated at least three times and the the data were reproducible.
Randomization	Cells were plated and distributed at equal density for treatment and control groups. The confluence of the cells at the time of treatment was noted to be equal and the allocation of treatment was randomly assigned.
Blinding	No blinding experiments.

Reporting for specific materials, systems and methods

We require information from authors about some types of materials, experimental systems and methods used in many studies. Here, indicate whether each material, system or method listed is relevant to your study. If you are not sure if a list item applies to your research, read the appropriate section before selecting a response.

Materials & experimental systems

n/a	Involved in the study	
☐	☒	Antibodies
☐	☒	Eukaryotic cell lines
☒	☐	Palaeontology
☒	☐	Animals and other organisms
☒	☐	Human research participants
☒	☐	Clinical data

Methods

n/a	Involved in the study	
☒	☐	ChIP-seq
☒	☐	Flow cytometry
☒	☐	MRI-based neuroimaging

Antibodies

Antibodies used	The detailed information of all antibodies used has been provided in Methods: ERK (CST, #9102); pERK (CST, #9101); GAPDH (Bioworld Technology, Nanjing, MB001); FLAG(Sigma, F1804); Anti-rabbit (LI-COR Biosciences, 926-32213); Anti-mouse (LI-COR Biosciences, 926-68022); HRP-conjugated anti-rabbit secondary antibodies (cwbiotech, Beijing, CW0103s). All primary antibodies for western were used at a dilution of 1:1000. All secondary antibodies were used at a dilution of 1:10000. Nestin (Aves Labs, 1:500), secondary antibody (Abcam, ab150169,1:500) were used for IF.
Validation	The antibodies were validated by western blot (correct molecular weight, expected response to physiological stimuli, overexpression and/or knockdown of the target).

Eukaryotic cell lines

Policy information about cell lines

Cell line source(s)	Wildtype mouse ESCs were previously generated by us (please see Wang Y. et al. Nature Genet 39.380-385 (2007)). And HEK293T cells were from ATCC.
Authentication	RNA expression by RT-qPCR was used to confirm wild type, knockdown, knockout or overexpression cells. No other cell line authentication were performed.
Mycoplasma contamination	All cell lines tested negative for mycoplasma contamination.
Commonly misidentified lines (See ICLAC register)	No such cell lines.
